# Harshness and unpredictability during childhood: an approach from life-history theory to understanding risk behaviors

**DOI:** 10.3389/fpsyg.2025.1624659

**Published:** 2025-10-16

**Authors:** Eugenio J. Guzmán-Lavín, Oriana Figueroa, José Antonio Muñoz-Reyes, Nerea Aldunate, Pablo Polo

**Affiliations:** ^1^Laboratorio de Comportamiento Animal y Humano, Centro de Investigación en Complejidad Social, Facultad de Gobierno, Universidad del Desarrollo, Santiago, Chile; ^2^Facultad de Salud y Ciencias Sociales, Universidad de las Américas, Santiago, Chile

**Keywords:** life-history theory, risk propensity, early environment, life-history strategies, harshness, unpredictability

## Abstract

**Introduction:**

Previous studies have investigated the relationship between childhood experiences of harshness and unpredictability and risky adult behaviors from a life-history theory perspective. However, findings have been inconsistent, suggesting that the relationship between early environments and current behavior is complex and may be influenced by moderating variables. This study examined whether childhood harshness (resource scarcity) and unpredictability (proximal environmental instability) were positively related to risk propensity, considering reproductive strategy-related trade-offs (i.e., the age of first sexual intercourse and the age of menarche) and current environmental factors (i.e., being in a committed relationship, perceived family support, and poverty rate of the participant's municipality) as potential moderators.

**Methods:**

We sampled 368 individuals in two settings: college classrooms and a controlled laboratory environment.

**Results:**

Overall, we did not find a clear relationship between perceived childhood environment and risk-taking. Contrary to expectations, we found a positive relationship between perceived childhood harshness and risk propensity in women who delayed their first sexual intercourse. Exploratory analyses by data collection setting revealed that harsh and unpredictable childhood environments may impact risk propensity differently, though no coherent pattern emerged.

**Discussion:**

This study underscores the importance of context dependence and the need to consider additional variables that may moderate the relationship between childhood experiences and risk-taking behaviors.

## 1 Introduction

Risk-taking behaviors involve actions or inactions with a degree of danger or potential harm, yet they also offer the chance of gaining rewards in the short or long term (Leigh, 1999). Research has suggested that variations in risk propensity are influenced by specific ecological factors that determine how individuals allocate their resources (Griskevicius et al., 2011a). In this sense, life-history theory is a conceptual framework that aims to explain how natural selection has favored life-history strategies that balance resource allocation in response to environmental conditions and evolutionary pressures to optimize the survival and reproduction of the organisms (Stearns, 1992). Life-history theory has been extensively applied across various taxa, including humans, to understand risk-taking behaviors, with studies examining domains such as financial risk (e.g., Griskevicius et al., 2011a, 2013; Amir et al., 2018; Martinez et al., 2022), health-related risk (e.g., Chang and Lu, 2018; Mishra et al., 2017), and social or reproductive risk (e.g., Figueredo et al., 2007; Salmon et al., 2009; Simpson et al., 2012). Despite this scope, evidence remains mixed and often inconclusive, particularly regarding how childhood harshness (e.g., resource scarcity) and unpredictability—operationalized as proximal environmental instability, referring to frequent and unpredictable changes in the child's immediate environment (e.g., unpredictability related to the family routines)—influence financial risk-taking. These childhood factors are central in life-history theory, as they shape life-history strategies by signaling resource availability and environmental stability, yet their specific effects on financial risk propensity remain underexplored with mixed evidence. Moreover, few studies have employed behavioral measures of risk-taking, such as the Balloon Analog Risk Task (BART), while systematically examining how these childhood conditions interact with reproductive strategy-related trade-offs (e.g., age at first sexual intercourse, age at menarche) and current environmental factors (e.g., socioeconomic conditions, social support). This leaves a gap in understanding how past childhood conditions and present contexts jointly shape risk behaviors. This study addresses this gap by investigating how childhood harshness and unpredictability influence behaviorally assessed financial risk propensity, considering both reproductive trade-offs and current environmental factors as moderators.

In the context of life-history theory, the early environment—comprising the physical, ecological, and social conditions from birth to early childhood (Klimek et al., 2023)—is a critical determinant of developmental trajectories. It plays a central role in shaping resource allocation, defining essential developmental periods, and calibrating adaptive responses to environmental signals, including risk-taking behaviors (Del Giudice, 2018; Doom et al., 2016). In particular, two salient dimensions—harshness and unpredictability—have been identified as key influences on the development of individual life-history strategies (Ellis et al., 2009; Klimek et al., 2023). Harshness reflects extrinsic morbidity-mortality caused by largely uncontrollable factors and can manifest as threat (e.g., violence, predation) or deprivation (e.g., resource scarcity), each potentially exerting distinct effects on behavior (Fenneman and Frankenhuis, 2020; Ellis et al., 2022). Harshness quantifies the extent to which these extrinsic factors cause disability or lead to premature death at different ages within a population (Ellis et al., 2009). In humans, childhood harshness has commonly been measured through indicators of deprivation such as socioeconomic status (SES), parental education, neighborhood SES, and available resources (Maranges et al., 2022) or indicators of threat and violence as emotional, physical and sexual abuse or emotional and physical neglect (Bernstein et al., 1994). Unpredictability denotes uncertainty in the environment and may reflect stochastic variation in harshness (e.g., random economic shifts; Doom et al., 2016) or proximal instability in family dynamics (Frankenhuis and Del Giudice, 2012), such as residential changes, parental job changes, parental transitions, inconsistencies/instabilities, among others (Maranges et al., 2022; Wu et al., 2020).

Previous studies have proposed that exposure to harshness and unpredictability during early life, including childhood and adolescence, may increase the propensity to take risks. Such tendencies have been documented across diverse domains, including health-related risks (Chang and Lu, 2018), financial and economic risks (Amir et al., 2018; Griskevicius et al., 2011a, 2013), and other risk-related behaviors (for a review, see Wu et al., 2020). This propensity is thought to be not merely a byproduct of adversity, but an adaptive response. It may trigger the adoption of strategies that prioritize impulsive, short-term decisions over deliberative, long-term ones (Mishra et al., 2017), to increase the chances of obtaining resources crucial for short-term survival and reproduction (Chang and Lu, 2018). Research by Griskevicius et al. (2011a, 2013), operationalizing harshness as deprivation (i.e., low resource availability) and employing behavioral measures of financial risk-taking, supports this idea. Their findings indicate that individuals from deprived (harsh) backgrounds are more likely to engage in riskier behaviors, particularly in the presence of strong mortality cues such as violence or death. This suggests that current conditions (e.g., mortality cues highlighted in the experiment as a form of threat-related harshness) moderate the relationship between childhood environment and risk-taking behaviors. Nevertheless, some findings challenge the positive relationship between childhood adversity and risk-taking in financial scenarios. For instance, Amir et al. (2018), also using a deprivation-based harshness measure (SES), found a pattern of risk aversion (rather than risk propensity) among participants raised in deprived settings, suggesting that in such environments, avoiding economic losses is crucial for welfare. Martinez et al. (2022) found no evidence linking childhood harshness or unpredictability (measured as deprivation and proximal environment and family unpredictability) with risk propensity in later life. Furthermore, Pepper et al. (2017), in replicating Griskevicius et al. (2011b), failed to find the interaction between childhood SES and mortality-priming conditions with risk-taking.

Given the mixed evidence described above, it is crucial to consider other aspects of life-history theory—such as the trade-off between somatic investment and reproduction—, and to examine current environmental factors as potential moderators of the relationship between early environments and risk-taking behaviors. Early environments characterized by harshness or unpredictability can influence reproductive strategy-related trade-offs, such as the onset of menarche (Culpin et al., 2014) and the initiation of sexual activities (Alvergne et al., 2008; Sheppard and Sear, 2012). Such experiences could predispose individuals to maximize short-term reproductive success, at the expense of investments in long-term development (Ellis et al., 2009). Short-term strategies may arise in response to morbidity-mortality cues, which have been associated with increased risk-taking behavior. This aligns with findings from Griskevicius et al. (2011a, 2013). While life-history theory posits that early environments covary with sexual debut and the onset of menarche—shaping strategies along a fast-slow continuum—, recent critics argue that human life-history strategies are context-dependent and plastic. These strategies can recalibrate throughout development, shaped by the interaction between past experiences and current environmental conditions (Del Giudice, 2018; Nettle and Frankenhuis, 2020). In this sense, the association between harshness or unpredictability and risk-taking behaviors may be more salient in individuals who exhibit life-history traits associated with short-term reproductive strategies.

In the same vein, current environmental factors could moderate the relationship between early environments and risk-taking behaviors. While previous research has acknowledged the role of current environmental factors, such as individual socioeconomic status (i.e., current harshness) in shaping the relationship between childhood harshness and risk propensity, the results have been mixed (Amir et al., 2018; Griskevicius et al., 2011a, 2013; Pepper et al., 2017). It remains unclear how current environmental unpredictability (e.g., being in a committed relationship and family support) and current harshness (e.g., neighborhood socioeconomic status) may impact this dynamic. Some authors suggest that being in a committed relationship can have a stabilizing effect through supportive social bonds, which may reduce the inclination toward risk-taking behaviors (Sevi, 2019; Silva Júnior et al., 2022). Similarly, Mohammadpoorasl et al. (2013) emphasize the protective role of family support in preventing engagement in risk-taking behaviors. Furthermore, living in socioeconomically deprived neighborhoods has been linked to higher environmental harshness (Nettle, 2010), which in turn has been associated with an earlier age at first birth, lower birth weights, and earlier age at menarche, among other characteristics (Nettle et al., 2011; Sung et al., 2016). However, the role of current environmental factors—such as relationship status, family support, and neighborhood context—as moderators in the relationship between childhood conditions and risk propensity remains relatively unexplored.

While informative, the existing body of evidence remains inconclusive and limited. This highlights the need for research that not only considers the lasting impact of childhood conditions on risk-taking behaviors, but also examines how life-history traits and current environmental factors act as crucial moderators. This approach offers a more comprehensive understanding of the variables that influence individual risk-taking, integrating prior influences with present circumstances. In addition, several methodological limitations persist, including small sample sizes (Griskevicius et al., 2011a, 2013) and reliance on online surveys (Amir et al., 2018), which could affect the reliability and validity of the collected data (see Saleh and Bista, 2017). These methodological challenges suggest that previous findings might not fully capture the influence of childhood environments on risk-taking behaviors.

Building upon current knowledge and addressing existing challenges, this study aims to understand, within the framework of life-history theory, the impact of childhood harshness— operationalized as childhood deprivation—and unpredictability—operationalized as proximal environmental and family instability—on adult financial risk-taking. Considering the complexity of these relationships, we examined the trade-off between development and reproduction—central to life-history strategies—as reflected in the age at first sexual relationship and the onset of menarche. We also considered current environmental factors, including neighborhood socioeconomic status, being in a committed relationship, and family support, as potential moderators. More specifically, resource deprivation—signaling extrinsic scarcity—may adaptively increase risk-taking in an effort to secure resources, particularly when reproductive strategies prioritize short-term gains (e.g., early sexual debut or menarche) or when current deprivation persists (e.g., low neighborhood SES). Proximal instability, reflecting disrupted conditions in environmental and family contexts, may increase risk-taking among individuals raised in unstable environments, whereas stabilizing current factors (e.g., being in a relationship or receiving family support) could shift this tendency toward risk-aversion. Based on this rationale, we proposed the following specific predictions: (1) individuals exposed to harsh and unpredictable childhood environments and who experience early initiation of sexual activity and menarche will exhibit an elevated inclination for risk-taking behaviors; and (2) individuals exposed to harsh and unpredictable childhood environments but currently living in a higher socioeconomic neighborhood, engaging in stable relationships, or having robust family contact/support will display reduced inclinations toward risk-taking behaviors.

## 2 Materials and methods

### 2.1 Participants

The initial participant pool consisted of 405 individuals. However, the final sample was reduced to 368 due to missing data and the exclusion of one participant with atypical task engagement. Specifically, eight participants were excluded due to the absence of sex information. Twenty-one participants were excluded due to missing questionnaire responses, and seven more due to missing Balloon Analog Risk Task (BART) scores. Finally, one participant was excluded because of anomalous BART performance, having experienced only one balloon explosion across 30 trials, which suggested atypical task engagement. Participants ranged in age from 18 to 58 years (*M* = 24.52, *SD* = 6.71), and 195 identified as female.

This study was part of a larger research project. As a result, participants were recruited in two different contexts: (1) data collection conducted in classrooms across various undergraduate programs at Universidad del Desarrollo between August and October 2022 (*n* = 179); and (2) a laboratory-based data collection conducted at the Laboratorio de Comportamiento Animal y Humano, Universidad del Desarrollo, between May and August 2023 (*n* = 189). All participants received a compensation of $15,000 Chilean pesos (approximately USD $15.04) for their participation in the study. Participants were informed that part of their payment would depend on their performance in the BART; however, for ethical reasons, all received the full compensation at the end of the procedure.

### 2.2 Procedures

In both data collection contexts, participants provided written informed consent (IC) prior to beginning the study. Following the IC briefing, participants completed a set of questionnaires including all of those employed in this study. Before the participants received instructions for the BART, they played an economic game (not employed in this study) in groups. The outcome of this game was not known until the end of the procedure, so it is unlikely to influence decisions in the BART. Next, they individually played the BART. Finally, they made public donations to an NGO as a part of the larger research project. In the data collection conducted in the field, the tasks were completed in classrooms (~25 students). During each session, a team of five researchers was present to ensure accurate task completion. Additionally, students were informed beforehand to bring a computer to respond to the tasks. The research team supplied a computer for those lacking one or experiencing technical difficulties. The laboratory-based data collection was supported by at least three researchers and involved groups of four to six participants who completed the tasks individually in separate and isolated rooms provided with a computer. Both the questionnaires and the BART (Lejuez et al., 2002) were run on the Inquisit software (Millisecond Software, 2022a,b), through an online link (Inquisit 6 Web), in the case of the field sample, or by running locally the script on the laboratory computers (Inquisit Lab 5). Although the procedural and instrumental consistency was maintained across both data collections, the separation in time and the recruitment in different contexts necessitated the consideration of potential “context” effects (see Statistical Analyses).

### 2.3 Measures

#### 2.3.1 Harshness and unpredictability

Harshness (resource deprivation) and unpredictability (proximal environmental and family instability) were assessed using a questionnaire developed by Maranges et al. (2022), designed to measure perceptions of childhood experiences in these domains. In this framework, harshness refers to growing up in a context of limited monetary and material resources, reflected in difficulties affording discretionary or even basic items and experiences (e.g., family unable to afford luxury goods, restaurant meals, new clothes, or holiday gifts), as well as perceptions of living in a relatively poor household or neighborhood. Unpredictability refers to frequent changes or inconsistencies in the immediate family environment, such as uncertainty about caregiver presence (e.g., who would pick the child up from school), instability in relationships between caregivers, frequent moves between homes or schools, changes in household composition, and chaotic or irregular daily routines. This instrument is divided into two scales, one evaluating childhood harshness with 11 items with Cronbach's α = 0.82, and the other assessing unpredictability with 15 items with Cronbach's α = 0.93. Both scales employ a 7-point Likert scale ranging from 1 (strongly disagree) to 7 (strongly agree). This questionnaire had not been previously validated for the Chilean population or for any other Spanish-speaking population. Therefore, we translated the original items into Spanish and checked that the items were understood and comprehended in the same way as the original ones. In doing this, we performed a pilot phase in which both academic professionals and university students evaluated the clarity and comprehension of each item. Feedback from this pilot was incorporated to ensure semantic equivalence and cultural appropriateness of the translated instrument. Our reliability analysis mirrored the questionnaire's high internal consistency, yielding alpha values of 0.91 for harshness and 0.89 for unpredictability.

#### 2.3.2 Family support

To evaluate the participants' degree of current family support, we utilized the family social contact/support subscale (items 13, 14 and 15) from the Mini-K scale (Figueredo et al., 2006). The Mini-K, a concise 20-item self-report instrument, assesses various domains related to life-history strategies. Our focus on the mentioned subscale aimed to capture the essence of familial support, given its specific relevance to social contact and support mechanisms within the family context. The subscale demonstrated high reliability in our analysis, with Cronbach's α = 0.85, indicating strong internal consistency.

#### 2.3.3 Sociodemographic, life-history reproductive strategy-related trade-offs and current environmental factors

We employed single questions to capture sociodemographic variables, life-history reproductive strategy-related trade-offs, and current environmental factors. These included participant sex, age, municipality of residence, age at menarche, relationship status, and age at first sexual relationship. We assessed neighborhood SES as an indicator of participants' SES matching participants' municipality of residence with the “Percentage of People in Poverty by Income” in that municipality (Ministerio de Desarrollo Social, 2022), creating a variable named “poverty rate by municipality”. Furthermore, we defined relationship status through a binary variable, with “1” indicating the presence and “0” indicating the absence of a committed relationship. For participants yet to experience sexual intercourse, we assigned “NA” to their age at first sexual intercourse.

#### 2.3.4 Risk-taking behavior

To measure risk-taking behavior, participants played a modified version of the Balloon Analog Risk Task (BART), a computerized task designed by Lejuez et al. (2002). Participants were instructed to inflate a simulated balloon by clicking on a pump button. Each pump increases the size of the balloon and adds 5 cents to a temporary bank and also increases the probability that the balloon explodes. Participants can stop inflating at any point and click on a button labeled “Collect $$$” to transfer the accumulated money from the temporary bank to a “permanent bank”. If the balloon explodes, the participant loses all the money from the temporary bank. In the modified version of the BART used in this study, a total of 30 red balloons were presented instead of the color variety in the original version. The calculation of the explosion probability was held constant to simplify the task and focus on the individual's overall risk propensity, rather than adaptation to different probabilities associated with different balloon colors. Each red balloon still had a unique explosion probability, which was calculated by randomly selecting a number without replacement from a series of integers from 1 to 128. If the number 1 is selected, the balloon explodes with a distinctive “pop” sound. Accordingly, the initial pump held a 1/128 probability of causing an explosion. This probability rose incrementally: 1/127 on the second pump, 1/126 on the third, and so on, culminating in a certainty of explosion (a probability of 1/1) by the 128th pump. The probability of explosion was unknown to the participants, but they were informed that the balloon could explode either on the first pump or after making enough pumps to fill the screen—i.e., participants were aware that with each additional pump, the probability of the balloon exploding increased, like in real life scenarios. We changed the dollar amount to Chilean pesos, and each “pump” given to the balloon delivered 5 Chilean pesos. In our study, consistent with the methodology proposed by Lejuez et al. (2002), the primary dependent measure on the BART was the adjusted average number of pumps. This adjusted value is defined as the average number of pumps on balloons that did not explode, which is preferable to the unadjusted average measure because the number of pumps is necessarily constrained on balloons that exploded, thereby limiting between-participant variability in the unadjusted averages (see Aklin et al., 2005). Additionally, we considered other risk-taking indices that have been tested in the literature for conducting complementary and exploratory analysis (e.g., Griskevicius et al., 2013; see Supplementary Tables 3–6).

### 2.4 Data analyses

#### 2.4.1 Descriptive analysis

Means and standard deviations were obtained for all continuous variables. Given that we dealt with data collected in different contexts, we employed Wilcoxon rank sum tests to explore possible differences between them. Overall, we found that participants from both contexts differed in many of the variables we measured including scores in the BART. Therefore, we created a dummy variable named “Context” to control for differences in the BART scores in each model. This variable distinguishes between the two data collection contexts, allowing for the examination of context-specific effects on the dependent variable and ensuring that the findings were reflective of the investigated phenomena rather than biases of temporal variance. Following these analyses, we analyzed the relationships among the different variables of interest using Spearman's correlation coefficients. To ensure consistency in the sample across all correlations, we applied listwise deletion, removing cases with missing values before computing the correlation matrix. First, we applied listwise deletion to the global/general sample without including the age at first sexual intercourse variable. Then, we repeated the analysis including this variable.

#### 2.4.2 Regression models

In our statistical models, we employed robust linear models (RLM) using MM-estimation via the “lmrob” function in the “robustbase” package in R (Maechler et al., 2024). This choice was driven by the presence of potential outliers in our data (see Supplementary Section 3), which robust regression techniques are adept at handling (Greco et al., 2019). The MM-estimator used in our RLM has a high breakdown point, allowing it to tolerate numerous outliers without producing biased estimates (Aleng et al., 2020). Thus, RLM's were crucial for ensuring valid regression analyses by minimizing the influence of deviant data points, which can be particularly problematic in ordinary least squares regression (Greco et al., 2019). Given the natural variability in our data, this robustness was essential.

To test our first prediction, first, we fit a basal model considering only the effects of perceived childhood harshness and unpredictability on risk propensity. Then, we examined main and moderating effects of age at first sexual relationship, and subsequently, in a model restricted to female participants, we incorporated the age of menarche. Since sexual orientation may influence the age at first sexual relationship (e.g., Tornello et al., 2014; van Griensven et al., 2004), we tested for differences between heterosexual (*n* = 286) and non-heterosexual (including bisexual, *n* = 46) individuals in our sample. Despite that the mean age of sexual debut was lower for non-heterosexual individuals (*M* = 16.4, *SD* = 2.25) compared to heterosexual individuals (*M* = 17.2, *SD* = 2.73), the difference is not statistically significant (parametric: *t* = 1.88, *df* = 326, *p* = 0.060; non-parametric: *U* = 5117, *p* = 0.117). To address our second prediction, we fit a model considering main and moderating effects of (1) the poverty rate by municipality; (2) being (or not) in a committed relationship (dummy variable); and (3) family contact/support (i.e., current environmental factors) in the relationship between childhood harshness and unpredictability on risk-taking behaviors. Age, sex, and context-based data collection were considered as control variables in all the models. In addition, in the Supplementary Section 1), we carried out these same analyses, but separating the samples according to the data collection context, in order to explore the possible effects that might arise.

Prior to RLM analyses, we standardized continuous variables to z-scores. All analyses and graphs were performed using R version 4.3.2. Correlation graphs were performed with “corrplot” package and moderation graphs were performed with “ggplot2” (Wickham, 2016) and “Hmisc” (Harrell, 2023) packages. The level of significance was set at α =0.05.

#### 2.4.3 Sensitivity power analysis

Although this study was part of a larger project, and therefore the scope of the sample was limited from the beginning, different sensitivity power analyses were carried out using the G^*^Power 3.1.9.6 software. These analyses were conducted for the different regression models used to test our hypotheses, keeping a constant alpha of 0.05 and a statistical power of 0.80. For prediction one, which considered a sample of 330 participants, 6 predictors, and 2 interaction terms, the sensitivity analysis indicated a capability to detect a minimum effect size (*f*^2^) of 0.05, which is considered a small to medium effect. For prediction one, but considering only female participants, entailing a sample of 173 participants, 6 predictors, and 4 interaction terms, the sensitivity analysis indicated a capability to detect a minimum effect size (*f*^2^) of 0.10. Finally, for prediction two, considering a sample of 362 participants, 8 predictors, and 6 interaction terms, the sensitivity power analysis indicated a capability to detect a minimum effect size (*f*^2^) of 0.05.

## 3 Results

### 3.1 Descriptive analysis

Our descriptive analysis uncovered significant variations in the means of six out of the eight variables examined, reflecting differences across both data collection contexts (Table 1). Specifically, the means and standard deviations for each variable were calculated for the overall sample, as well as separately for each data collection context. The table also includes Wilcoxon Rank Sum test comparing the means between contexts, indicating where significant differences were found.

**Table 1:** Means and standard deviations by context.

**Variable**	**General**	**Context 0**	**Context 1^a^**	**Wilcoxon rank sum test (Context 0 and 1)**	**Observed range**	**Possible range**
Age	24.52 (6.71)	21.15 (2.30)	27.71 (7.86)	5834^***^	18–58	–
Age at first sexual relationship	17.1 (2.68)	16.86 (1.93)	17.30 (3.16)	13306	11–32	–
Age at menarche	12.16 (1.54)	12.40 (1.57)	11.98 (1.49)	5540.5	8–17	–
Unpredictability	2.27 (1.09)	2.11 (0.98)	2.42 (1.17)	14397^*^	1–6.2	1–7
Harshness	3.71 (1.50)	3.08 (1.26)	4.31 (1.46)	8971^***^	1–7	1–7
Family support	4.85 (1.70)	5.06 (1.67)	4.66 (1.71)	19334^*^	1–7	1–7
Risk propensity	40.26 (16.16)	43.33 (14.70)	37.35 (16.97)	19204^*^	4.7–94.4	–
Poverty rate by municipality^b^	0.06 (0.03)	0.05 (0.02)	0.06 (0.03)	11584^***^	0.027–0.153	0–1.0

Means are presented with standard deviations in parentheses. Wilcoxon rank sum tests compare variables between Context 0 and Context 1. Observed range reflects the minimum and maximum values within the current sample. Possible range corresponds to the theoretical range of each instrument or variable when applicable. For age-related variables (e.g., Age, Age at menarche), no theoretical range is specified. Ranges are based on mean scores for Harshness, Unpredictability, Family Support, and Risk Propensity.

^a^Context 1 refers to the laboratory-based sample.

^b^Poverty rate by municipality is in percentage.

^*^p < 0.05; ^***^p < 0.001.

### 3.2 Correlational analyses

**Figure 1:**
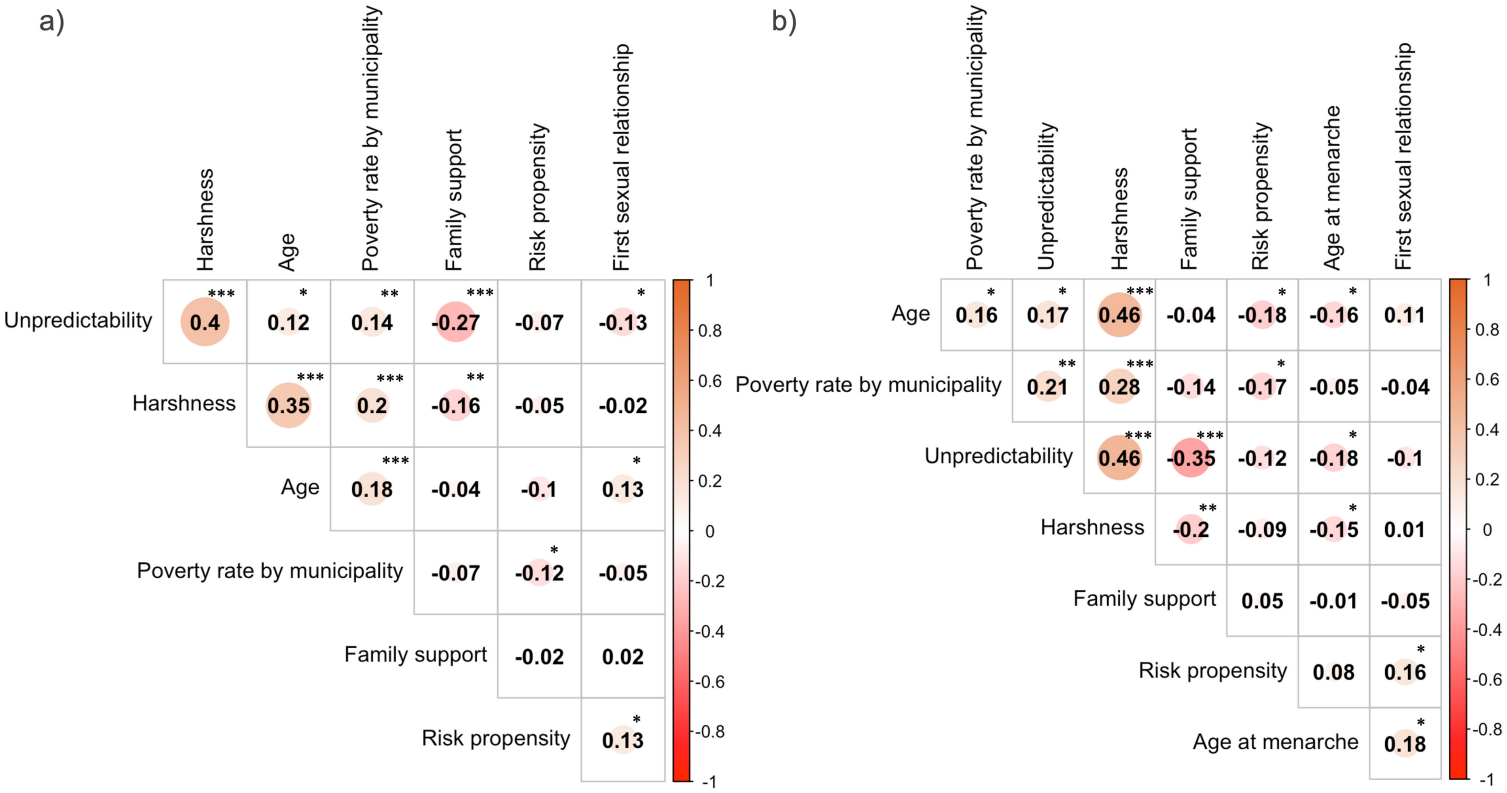
**(a)** Shows the correlations between the main variables used in the study for the whole sample, and **(b)** shows the correlations for the women-only sample. For correlations involving the variable “First Sexual Relationship”, the sample size was smaller: in **(a)**, *N* = 328 (instead of 366); in **(b)**, *N* = 171 (instead of 194). *p < 0.05; **p < 0.01; ***p < 0.001.

The correlation matrix (Figure 1a) delineates the relationship between the primary variables. Regarding the age at menarche, the correlations of interest are in Figure 1b.

### 3.3 Regression models

To test our first prediction, specifically, whether reproductive strategy-related trade-offs moderate the relationship between perceived childhood harshness and unpredictability with risk-taking behaviors (see Table 2), we fit three models. The first model serves as a baseline illustrating the relationship between childhood harshness and unpredictability with risk propensity, controlling for participants' age, sex, and data collection context. Overall, neither childhood harshness (β = 0.02, *SE* = 0.06, *p* = 0.730) nor unpredictability (β = −0.05, *SE* = 0.06, *p* = 0.334) were significantly associated with risk propensity. We only found significant results for sex and data collection context. That is, being a woman is related to a decrease of 0.40 standard deviation in risk propensity compared to men (β = −0.40, *SE* = 0.10, *p* < 0.001; *M*_*women*_ = 36.9, *M*_*men*_ = 43.4). In addition, belonging to the laboratory context is related to a decrease of 0.38 standard deviation in risk propensity compared to the field context (β = −0.38, *SE* = 0.12, *p* =0.003; *M*_*laboratory*_ = 37.0, *M*_*field*_ = 43.0). The second model extends this baseline by incorporating the age of the first sexual relationship and their interaction terms with childhood harshness and unpredictability. First, the age of the first sexual relationship shows a nearly significant positive relationship with risk propensity (β = 0.12, *SE* = 0.06, *p* = 0.051); however, this relationship is opposed to what was predicted. And second, both interaction terms were not significant (see Table 2, Model 2).

**Table 2:** Moderating effects of reproductive strategy-related trade-offs on risk propensity.

**Variables**	**Dependent variable: Risk propensity**		
	**(1)**	**(2)**	**(3)**
Constant	0.39^***^ (0.09)	0.41^***^ (0.10)	0.12 (0.13)
Sex (1)^a^	−0.40^***^ (0.10)	−0.45^***^ (0.11)	
Age	0.02 (0.07)	−0.03 (0.07)	−0.04 (0.09)
Context (1)^b^	−0.38^**^ (0.12)	−0.36^**^ (0.13)	−0.66^***^ (0.18)
Harshness	0.02 (0.06)	0.04 (0.07)	0.11 (0.08)
Unpredictability	−0.05 (0.06)	−0.02 (0.06)	−0.05 (0.07)
Age at first sexual relationship		0.12 (0.06)	0.19^*^ (0.07)
Age at menarche			0.04 (0.09)
Harshness ^*^ age at first sexual relationship		0.04 (0.08)	0.16^*^ (0.07)
Unpredictability ^*^ age at first sexual relationship		−0.02 (0.07)	0.06 (0.09)
Harshness ^*^ age at menarche			0.01 (0.09)
Unpredictability ^*^ age at menarche			−0.12 (0.08)
Observations	368	330	173
*R* ^2^	0.07	0.10	0.16
Adjusted *R*^2^	0.06	0.08	0.11
Residual Std. error	0.99 (*df* = 362)	0.97 (*df* = 321)	0.97 (*df* = 162)

The table presents the estimated beta coefficients (β) from the regression analysis. Standard errors are reported immediately below each coefficient in parentheses.

^a^Sex (1) corresponds to women.

^b^Context (1) corresponds to laboratory sample.

^*^p < 0.0**5;** ^**^p < 0.01; ^***^p < 0.001.

**Figure 2:**
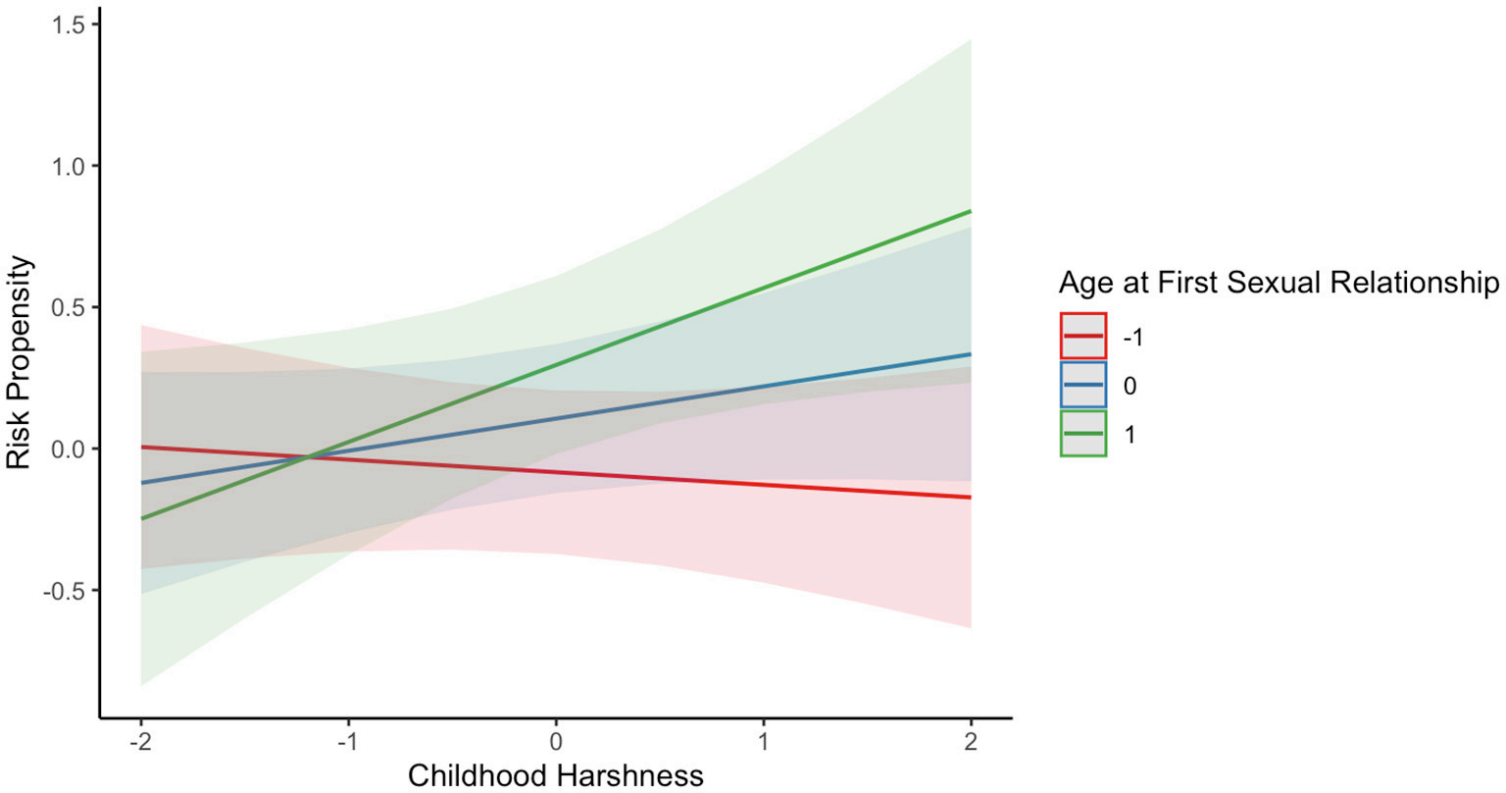
The figure illustrates the marginal effects of perceived childhood harshness on risk propensity at different levels of age at first sexual relationship in women. The relationship is depicted at three levels of age at first sexual relationship: the mean (blue line), one standard deviation above the mean (green line), and one standard deviation below the mean (red line).

The third model narrows its focus to women and incorporates the age at menarche as a moderator variable. However, this variable does not significantly influence the relationship between childhood harshness or unpredictability with risk propensity (see Table 2, Model 3). Instead, there was a positive and significant main effect of the age of the first sexual relationship (β = 0.19, *SE* = 0.07, *p* = 0.011). That is, those women who delayed their sexual debut were more prone to take risks, conversely to our prediction. Moreover, it was found that this variable moderates the relationship between childhood harshness and risk propensity (β = 0.16, *SE* = 0.07, *p* = 0.028), showing that the impact of childhood harshness on risk propensity was more relevant in women who delayed their first sexual relationship (see Figure 2).

Regarding our second prediction, we ran a robust linear regression model that included three moderators derived from the participants' current environmental factors (Table 3). None of the current environmental variables considered, that is, family support, poverty rate by municipality, and being in a committed relationship, were related to risk propensity, neither as a main effect nor in interaction with childhood harshness and unpredictability (see Table 3). Consistent with the models presented for prediction 1, sex (β = −0.40, *SE* = 0.11, *p* < 0.001) and context (β = −0.37, *SE* = 0.14, *p* = 0.007) were significant predictors of risk-taking following the same pattern of mean differences.

**Table 3:** Moderating effects of current environmental factors on risk propensity.

**Variables**	**Dependent variable: Risk propensity**
Constant	0.36^***^ (0.10)
Sex (1)^a^	−0.40^***^ (0.11)
Age	0.02 (0.07)
Context (1)^b^	−0.37^**^ (0.13)
Harshness	0.03 (0.09)
Unpredictability	−0.04 (0.08)
Family support	−0.04 (0.06)
Poverty rate by municipality	−0.09 (0.06)
Being in a committed relationship (1)^c^	0.02 (0.11)
Harshness ^*^ family support	−0.11 (0.07)
Unpredictability ^*^ family support	−0.03 (0.06)
Harshness ^*^ poverty rate by municipality	0.01 (0.06)
Unpredictability ^*^ poverty rate by municipality	−0.06 (0.05)
Harshness ^*^ being in a committed relationship(1)	0.04 (0.12)
Unpredictability ^*^ being in a committed relationship(1)	−0.03 (0.11)
Observations	362
*R* ^2^	0.10
Adjusted *R*^2^	0.06
Residual std. error	0.96 (*df* = 347)

The table presents the estimated beta coefficients from the regression analysis. Standard errors are reported immediately below each coefficient in parentheses.

^a^Sex (1) corresponds to women.

^b^Context (1) corresponds to laboratory sample.

^c^Being in a committed relationship (1) corresponds to being in a couple.

^**^p < 0.01; ^***^p < 0.001.

### 3.4 Exploratory regression models across data collection periods

Given that the data collection period was identified as a significant predictor across all models (Tables 2, 3) and that the samples differed in their sociodemographic and childhood characteristics (Table 1), exploratory robust linear regression models were conducted. These analyses stratified the sample according to each data collection period. The main findings are described below (for more details on the results, see Supplementary material, Supplementary Tables 1, 2, Supplementary Figures 1, 2).

Regarding the first prediction, we found that for women's sample from the laboratory setting, perceived harshness during childhood was positively associated with risk-taking (β = 0.21, *SE* = 0.1, *p* = 0.035), whereas perceived unpredictability during childhood was negatively associated with risk-taking (β = −0.23, *SE* = 0.09, *p* = 0.011; *R*-squared: 0.17; adjusted *R*-squared: 0.08, *n* = 97). We found no significant effects when considering men and women, either in the field or laboratory context.

In terms of prediction two, in the field sample, it was found that perceived childhood unpredictability was negatively associated with risk-taking in both men and women as a main effect (β = −0.22, *SE* = 0.09, *p* = 0.024; *R*-squared: 0.12; adjusted *R*-squared: 0.05, *n* = 174). In addition, the relationship between unpredictability and risk propensity was moderated by the poverty rate by municipality (β = −0.18, *SE* = 0.09, *p* = 0.036) and by being in a committed relationship (β = 0.50, *SE* = 0.15, *p* < 0.001). That is, for individuals living in municipalities with higher poverty rates, an increase in childhood unpredictability was associated with a decrease in risk propensity (see Supplementary Figure 1), and individuals in a committed relationship showed a positive relationship between unpredictability and risk propensity. In the laboratory sample, our results revealed that being in a committed relationship (β = −0.36, *SE* = 0.15, *p* = 0.022) was a significant moderator in the relationship between unpredictability and risk propensity (*R*-squared: 0.18; adjusted *R*-squared: 0.12, *n* = 188; see Supplementary Figure 2) but in the opposite direction compared to the field sample. This result indicates that for individuals who are in a committed relationship, an increase in unpredictability during childhood was associated with a decrease in risk propensity.

## 4 Discussion

Grounded in life-history theory, this study aimed to investigate the relationship between childhood conditions—marked by perceived harshness (resource deprivation) and unpredictability (proximal environmental and family instability)—and risk-taking behavior during adulthood, assessed via the balloon analog risk task. Specifically, we sought to understand how this association could be moderated by certain life-history reproductive strategy-related trade-offs and current environmental factors. Overall, our results showed that there was no direct relationship between childhood conditions and risk propensity. This finding was not totally unexpected since we predicted that this relationship could emerge when accounting for moderators. Nevertheless, even after adjusting for these factors, the expected relationship between childhood conditions and risk-taking behavior was not observed as predicted.

To contextualize our findings, it is crucial to consider how life-history strategies may influence the relationship between early experiences and risk-taking behaviors in adulthood. Life-history strategies can be considered as a set of coadapted traits involved in different resource-allocation trade-offs (Del Giudice et al., 2015). Based on this framework, early environmental factors are expected to influence demographic life-history reproductive strategy-related trade-offs such as the onset of sexual maturation (e.g., menarche) or the age of sexual debut (e.g., Alvergne et al., 2008; Sheppard and Sear, 2012). Additionally, the early environment may affect the expression of psychological traits and behaviors, including individual differences in risk-taking behaviors in adulthood (e.g., Ellis et al., 2022). However, recent critics argue that human life-history strategies are context-dependent and plastic, recalibrating across development based on interactions between past experiences and current conditions (Del Giudice, 2020; Nettle and Frankenhuis, 2020). In this sense, the effect of early experiences on risk propensity may depend upon the presence of this covariation with life-history reproductive strategy-related trade-offs. Accordingly, we predicted that individuals exposed to harsh and unpredictable childhood environments and who experienced early initiation of sexual activity and anticipated menarche would exhibit an elevated inclination toward risk-taking. This could reflect adaptive strategies selected to prioritize early reproduction in response to adverse childhood environments (Ellis et al., 2009; Griskevicius et al., 2011a). However, our robust regression models did not support this prediction. Indeed, contrary to our expectations, when including both age at first sexual intercourse and menarche, we found that women who had their sexual debut later were more prone to take risks. Furthermore, we found a moderation effect of sexual debut; that is, in women who delayed their first sexual relationship, there was a positive relationship between childhood harshness and risk-taking propensity. Once again, these results were opposite to our expectations.

This finding is difficult to explain given the previous evidence suggesting that delaying the first sexual encounter is associated with strategies related to the “slow” continuum of life-history strategies —which is associated with risk aversion behaviors and experiences of more predictable and/or favorable environments during childhood (see Ellis et al., 2022). It is thought that these environments reduce the costs associated with delaying the first reproduction (Belsky et al., 2012) given the absence of extrinsic mortalities and a greater predictability of the environment. Indeed, our results demonstrated that unpredictable environments were negatively related to the age of sexual debut, as expected by the life-history theory (Alvergne et al., 2008; Sheppard and Sear, 2012). Furthermore, childhood harshness and unpredictability were negatively related to the age of menarche. Thus, experiencing a more adverse childhood may have given signals aimed at maximizing reproductive success (in this case seen as anticipating the first sexual relationship or early initiation of puberty) later in adolescence; aligning with previous findings of life-history theory applied to humans (Alvergne et al., 2008; Ellis et al., 2009). Therefore, future studies are needed to understand the relationship between the onset of reproductive activity and risk behaviors and what variables not considered in this study might be affecting this relationship in order to understand our specific results.

Regarding our second prediction, we examined how current environmental factors influence the relationship between childhood environments and risk behaviors. Given the evidence that current cues of mortality risks are important to find the expected relationship between harsh and unpredictable childhood environment with risk-taking behaviors (Griskevicius et al., 2011a), we predicted that current environmental harshness (i.e., municipality poverty rate) and unpredictability (i.e., reduced family support and not being in a committed relationship) would moderate this relationship. However, our results did not support our predictions, as we failed to find any moderating effects (at least when considering the entire sample), despite that current SES was negatively correlated with risk propensity. In addition, neither unpredictability nor harshness during the present time were related to risk-taking behaviors. A potential explanation for our null results could be found in the Adaptive Calibration Model (ACM) of stress responsivity (Del Giudice et al., 2011). This model predicts a curvilinear pattern between early exposure to positive or negative environments and stress reactivity (Boyce and Ellis, 2005). Specifically, individuals who faced both favorable and unfavorable childhood environments could develop high reactivity profiles (higher sensitivity to stress; Ellis et al., 2009), which in turn would manifest in a propensity to take risks during adulthood (Del Giudice, 2011). This nonlinear relationship between childhood environment and risk-taking could explain the absence of clear results in our study and the importance of context dependence. Future studies considering the induction of stress could shed light on this nonlinear relationship. Furthermore, our findings' divergence from expectations may partly reflect the specific measures employed here: harshness as resource deprivation (e.g., limited family income) and unpredictability as proximal family instability (e.g., chaotic home life) per Maranges et al. (2022). Unlike Griskevicius et al. (2011a, 2013), which paired deprivation-based harshness (low SES) with effective mortality cues (e.g., violence priming) to increase risk-taking, our deprivation measure—without such cues—may not signal extrinsic mortality risks sufficiently to trigger short-term risk strategies in the BART's low-stakes context. Amir et al. (2018), also using deprivation-based SES and the BART, tested mortality cues but found no effect, with low SES linked to risk-aversion regardless of priming, mirroring our null direct effects and suggesting deprivation's risk signal weakens without threat reinforcement. For unpredictability, our proximal instability measure aligns with Martinez et al. (2022) null findings, contrasting with stochastic unpredictability's risk-taking link. Chronic family unpredictability might calibrate individuals toward caution in ambiguous tasks like the BART, unlike the impulsivity tied to unpredictable extrinsic changes. These operational distinctions—deprivation sans threat and proximal vs stochastic instability—highlight how specificity and context shape outcomes, potentially explaining our null and unexpected results.

### 4.1 Limitations

There are several limitations in this study. Firstly, our study used a sample of university students (field sample), and participants recruited through online public announcements (laboratory sample)—i.e., two distinct data collection contexts. This methodology could introduce biases into the sample, limiting the generality of the results to broader populations, because university students may not be representative of the general population (Hanel and Vione, 2016). Moreover, substantial baseline differences were observed between the two data collection contexts, which raises the possibility of unmeasured confounding variables—such as differences in socioeconomic background, cultural norms, or environmental factors—that could have influenced responses or task performance. While we statistically controlled for “context” in all models, we acknowledge that this approach cannot fully eliminate latent heterogeneity. To further assess robustness, we conducted stratified exploratory analyses by context. These analyses revealed some context-specific and sex-specific effects. For instance, harshness and unpredictability were positively and negatively related to risk-taking behavior, respectively, but only for women in the laboratory sample. In addition, an interaction between unpredictability and relationship status was found in both the laboratory and field context, but in opposite directions, and the interaction between unpredictability and rate of poverty was only found in the field context. More importantly, the interactions were not consistent with the predictions derived from the life-history theory. In sum, the exploratory analysis did not inform us about a coherent and clear context-specific pattern of results.

Secondly, our risk-taking propensity measure (i.e., the BART) accounts for financial risk-taking, disregarding different behavioral aspects related to risk propensity. Additionally, the low payment amounts can limit the applicability of the results to contexts where the decisions involved larger amounts, more comparable to significant real-life decisions (see Xu et al., 2018), and, accordingly, BART in our study could be perceived as a low-risk context. In future studies, the inclusion of multiple behavioral and self-report measures could strengthen the assessment of risk-taking, this was beyond the scope of the present study and should be considered in future research.

Thirdly, we did not capture important aspects of the participants' life histories, such as the number of children, the age at which they had their first child, and whether the participants' parents were separated during their childhood (unpredictability), among other variables. Although age at first sexual relationship is often used as a behavioral indicator of reproductive timing in life-history research, it is highly context-dependent and should be interpreted with caution. In our data, it showed a modest negative association with childhood unpredictability, but no association with childhood harshness as operationalized here (resource scarcity). This pattern suggests that sexual initiation may be more sensitive to environmental instability than to material deprivation, and/or that our harshness measure did not capture other adversity domains (e.g., neglect, violence, family dysfunction) that could be associated with earlier sexual initiation.

Fourthly, the poverty rate by municipality, used as an indicator of current environmental harshness, was relatively low and showed limited variability across our sample. Despite the fact that we found a significant interaction involving this variable in the exploratory analysis, future studies should include a more accurate measure of the current harshness, such as socioeconomic status, in order to capture a greater heterogeneity in this variable.

Finally, the questionnaires on perceived harshness and unpredictability during childhood, while capturing aspects such as the scarcity of resources (harshness), or stability (or not) in family support (unpredictability), do not consider other aspects like abuse, neglect, violence, neighborhood rate of criminality or family dysfunction, which could serve as additional predictors of risk propensity related to harshness and unpredictability. Moreover, these measures relied on retrospective self-reports referring to experiences before the age of 10; although this recall period was standardized to reduce ambiguity, such reports remain subject to potential recall bias. Additionally, while the Harshness and Unpredictability scales demonstrated high internal consistency in our sample, they have not undergone formal cultural validation for this context. The measures were translated and refined through a pilot process to ensure semantic clarity and cultural relevance, but we did not conduct back-translation or statistical procedures such as factorial invariance testing. Therefore, we cannot rule out the possibility that certain items may function differently across cultural contexts.

## 5 Conclusions

In the present study, we did not find a significant relationship between perceived childhood harshness (resource deprivation) and unpredictable and risk-taking propensity. This finding remained consistent even when incorporating variables related to reproductive strategy-related trade-offs and current environmental factors, suggesting that there may be other elements not considered in this analysis, such as sensitivity or reactivity to stress, nonlinear relationships, or other variables influencing this relationship. Our results suggest that life-history theory can provide a valuable framework for understanding human behavioral strategies, but that the relationship between early environments and current behaviors is more complex than expected and can be calibrated by several past and present factors.

## Data Availability

The datasets presented in this study can be found in online repositories. The names of the repository/repositories and accession number(s) can be found below: https://osf.io/zc6pa/?view_only=dce0c97fe7824fcbb47f1068637ed279.
