# Supplementary material for: Harshness and unpredictability during childhood: an approach from life-history theory to understanding risk behaviors
Source: Front Psychol. 2025 Oct 16;16:1624659. doi: 10.3389/fpsyg.2025.1624659 (PMC12571906; doi:10.3389/fpsyg.2025.1624659)
Supplement: Supplementary file 1 [file Supplementary_file_1.docx]

**Supplementary Materials**

**Section 1. Exploratory Regression Models Across Data Collection Contexts**

Given that the data collection context was identified as a significant predictor across all models (Tables 2 and 3) and that the samples differed in their sociodemographic and childhood characteristics (Table 1), exploratory robust linear regression models were conducted. These analyses stratified the sample according to each data collection context. The main findings are described below.

Regarding the first prediction, we found that for women’s sample from the laboratory setting, perceived harshness during childhood was positively associated with risk-taking (β = 0.21, SE = 0.1, p = .035), whereas perceived unpredictability during childhood was negatively associated with risk-taking (β = -0.23, SE = 0.09, p = .011; R-squared: 0.17; adjusted R-squared: 0.08, n = 97). We found no significant effects when considering men and women, either in the field or laboratory context.

**Table S1.** Moderating Effects of Reproductive Strategy-Related Trade-offs Across Data Collection Contexts for Women

|  | *Dependent variable* | |
| --- | --- | --- |
|  | Risk Propensity | |
|  | (Field sample) | (Laboratory sample) |
| Constant | 0.415** | -0.494*** |
|  | (0.155) | (0.115) |
| Age | 0.488** | -0.101 |
|  | (0.165) | (0.101) |
| Harshness | 0.120 | 0.209* |
|  | (0.157) | (0.097) |
| Age at first sexual relationship | 0.187 | 0.148 |
|  | (0.167) | (0.098) |
| Unpredictability | 0.183 | -0.228* |
|  | (0.121) | (0.088) |
| Age at menarche | -0.069 | -0.006 |
|  | (0.156) | (0.123) |
| Harshness * Age at first sexual relationship | 0.280 | 0.148^†^ |
|  | (0.188) | (0.088) |
| Unpredictability * Age at first sexual relationship | 0.063 | 0.074 |
|  | (0.133) | (0.131) |
| Harshness * Age at menarche | -0.199 | 0.136 |
|  | (0.187) | (0.099) |
| Unpredictability * Age at menarche | -0.054 | -0.154^†^ |
|  | (0.108) | (0.082) |
| Observations | 76 | 97 |
| R^2^ | 0.12 | 0.17 |
| Adjusted R^2^ | 0.003 | 0.084 |
| Residual Std. Error | 0.946 (df = 66) | 0.923 (df = 87) |

Note. Sex was not included in the models because both models account for only women ^†^p<0.1; *p<0.05; **p<0.01; ***p< 0.001.

Below, two robust regression models are presented (Table S2). Both models consider all variables included to test hypothesis two. In terms of prediction two, in the field sample, it was found that perceived childhood unpredictability was negatively associated with risk-taking in both men and women as a main effect (β = -0.22, SE = 0.09, p = .024; R-squared: 0.12; adjusted R-squared: 0.05, n = 174). In addition, the relationship between unpredictability and risk propensity was moderated by the poverty rate by municipality (β = -0.18, SE = 0.09, p = .036) and by being in a committed relationship (β = 0.50, SE = 0.15, p < .001). That is, for individuals living in municipalities with higher poverty rates, an increase in childhood unpredictability was associated with a decrease in risk propensity (see Figure S1) and individuals in a committed relationship showed a positive relationship between unpredictability and risk propensity. In the laboratory sample, our results revealed that being in a committed relationship (β = -0.36, SE = 0.15, p = .022) was a significant moderator in the relationship between unpredictability and risk propensity (R-squared: 0.18; adjusted R-squared: 0.12, n = 188; see Figure 4) but in the opposite direction compared to the field sample. This result indicates that for individuals who are in a committed relationship, an increase in unpredictability during childhood was associated with a decrease in risk propensity.

**Table S2.** Moderating Effects of Current Environmental Factors Across Data Collection Contexts

|  | *Dependent variable* | |
| --- | --- | --- |
|  | Risk propensity | |
|  | (Field sample) | (Laboratory sample) |
| Constant | 0.51*** | 0.13 |
|  | (0.14) | (0.15) |
| Sex(1) | -0.05 | -0.61*** |
|  | (0.14) | (0.15) |
| Age | 0.40** | 0.02 |
|  | (0.15) | (0.06) |
| Harshness | 0.21 | -0.06 |
|  | (0.12) | (0.15) |
| Unpredictability | -0.21* | 0.05 |
|  | (0.09) | (0.14) |
| Family Support | -0.01 | -0.10 |
|  | (0.10) | (0.09) |
| Poverty rate by municipality | 0.02 | -0.20* |
|  | (0.08) | (0.08) |
| Being in a committed relationship(1) | -0.10 | 0.01 |
|  | (0.18) | (0.17) |
| Harshness * Family support | -0.14 | -0.07 |
|  | (0.11) | (0.10) |
| Unpredictability * Family support | 0.001 | 0.004 |
|  | (0.06) | (0.09) |
| Harshness * Poverty rate by municipality | 0.06 | 0.05 |
|  | (0.11) | (0.08) |
| Unpredictability * Poverty rate by municipality | -0.18* | 0.04 |
|  | (0.09) | (0.07) |
| Harshness * Being in a committed relationship(1) | -0.18 | 0.23 |
|  | (0.20) | (0.18) |
| Unpredictability * Being in a committed relationship(1) | 0.50*** | -0.36* |
|  | (0.15) | (0.15) |
| Observations | 174 | 188 |
| R^2^ | 0.12 | 0.18 |
| Adjusted R^2^ | 0.05 | 0.12 |
| Residual Std. Error | 0.86 (df = 160) | 0.99 (df = 174) |
| *Note.* | *p<0.05; **p<0.01; ***p<0.001 | |


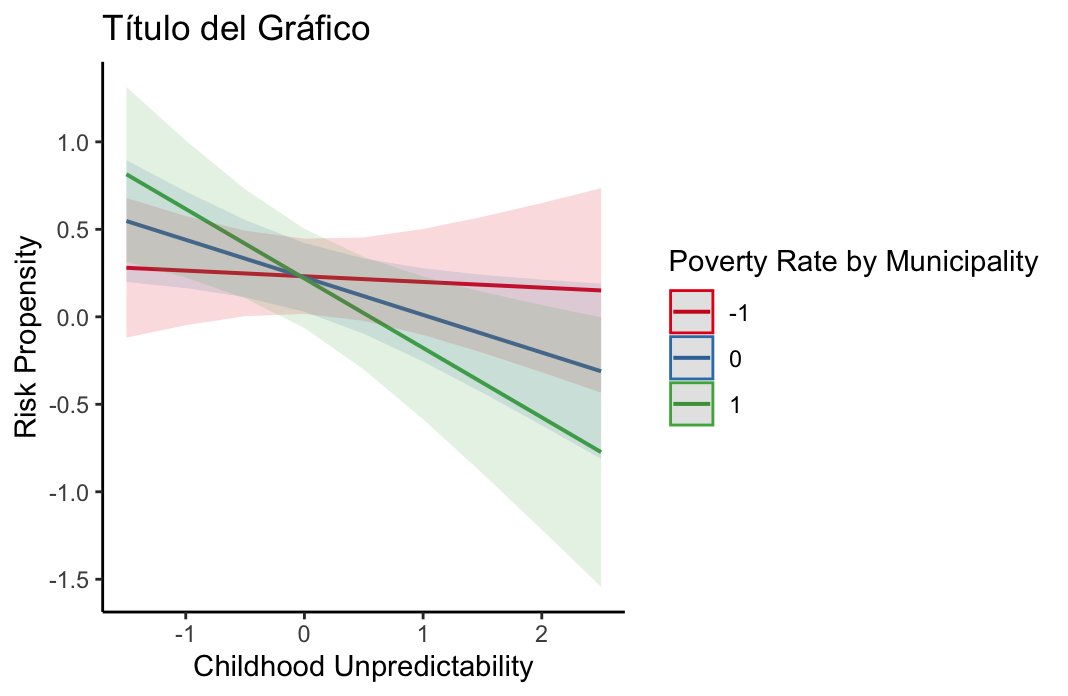


**Figure S1.** Marginal effects of perceived childhood unpredictability on risk propensity at different levels of the poverty rate by municipality.

Note. The relationship is drawn at three levels of poverty rate by municipality: the mean (blue line), one standard deviation above (green line) and one below the mean (red line).


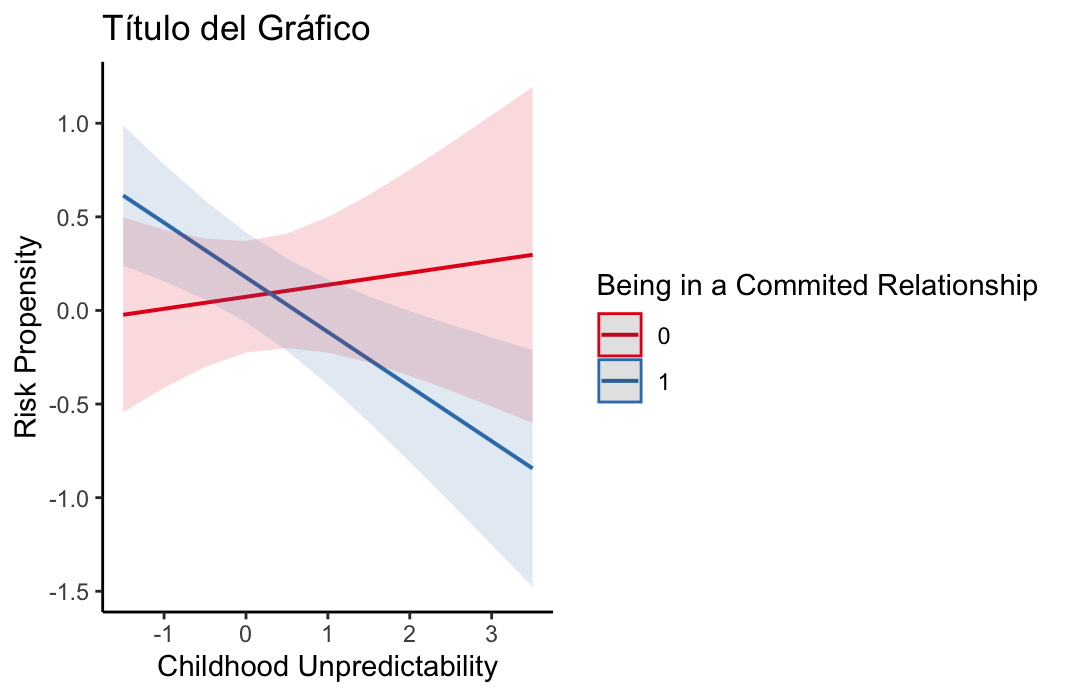


**Figure S2.** Marginal effects of how the relationship between childhood unpredictability and risk propensity varies at two distinct levels—being in a committed relationship (blue line) versus not being in one (red line; ceteris paribus).

**Section2. Robust Regression Models Using Other Indices from BART**

Below (Table S3 and S4), we present the same robust regression models that we used in tables 2 and 3 of the results section but changing the dependent variable. The dependent variable used was an adjusted measure of risk propensity used by Griskevicius et al. (2013). Such measure was created from the average of the standardized versions of (1) the average number of pumps per balloon and (2) the number of balloons exploded. It is important to mention that tables were only made for the models that include the entire sample.

**Table S3.** Griskevicius et al. (2013) Risk Propensity Measure for Hypothesis 1

|  | *Dependent variable* | | |
| --- | --- | --- | --- |
|  | Risk propensity | | |
|  | (1) | (2) | (3) |
| Constant | 0.389*** | 0.391*** | 0.120 |
|  | (0.092) | (0.097) | (0.115) |
| Sex(1) | -0.343*** | -0.388*** |  |
|  | (0.099) | (0.103) |  |
| Age | -0.025 | -0.077 | -0.109 |
|  | (0.065) | (0.066) | (0.088) |
| Context (1) | -0.386** | -0.347** | -0.567*** |
|  | (0.119) | (0.124) | (0.164) |
| Harshness | 0.038 | 0.055 | 0.149 |
|  | (0.058) | (0.062) | (0.076) |
| Unpredictability | -0.053 | -0.031 | -0.067 |
|  | (0.053) | (0.054) | (0.066) |
| Age at first sexual relationship |  | 0.122* | 0.202** |
|  |  | (0.057) | (0.066) |
| Age at menarche |  |  | 0.051 |
|  |  |  | (0.085) |
| Harshness * Age at first sexual relationship |  | 0.045 | 0.137* |
|  |  | (0.067) | (0.060) |
| Unpredictability * Age at first sexual relationship |  | -0.015 | 0.075 |
|  |  | (0.064) | (0.068) |
| Harshness * Age at menarche |  |  | 0.017 |
|  |  |  | (0.083) |
| Unpredictability * Age at menarche |  |  | -0.137 |
|  |  |  | (0.073) |
| Observations | 368 | 330 | 173 |
| R^2^ | 0.080 | 0.111 | 0.198 |
| Adjusted R^2^ | 0.067 | 0.089 | 0.148 |
| Residual Std. Error | 0.924 (df = 362) | 0.877 (df = 321) | 0.879 (df = 162) |
| *Note.* Similar to the authors, the risk measure was obtained by standardizing the number of pumps across the 30 trials (in the case of Griskevicius et al., 2013, there were 10 trials), then, the number of explosions was standardized for each participant. Finally, both standardized measures were averaged. *p<0.05; **p<0.01; ***p<0.001. | | | |

**Table S4.** Griskevicius et al. (2013) Risk Propensity Measure for Hypothesis 2

|  | *Dependent variable* |
| --- | --- |
|  | Risk propensity |
| Constant | 0.373*** |
|  | (0.102) |
| Sex(1) | -0.324** |
|  | (0.101) |
| Age | -0.018 |
|  | (0.062) |
| Context (1) | -0.368** |
|  | (0.123) |
| Harshness | 0.031 |
|  | (0.085) |
| Unpredictability | -0.016 |
|  | (0.079) |
| Family support | -0.057 |
|  | (0.057) |
| Poverty rate by municipality | -0.084 |
|  | (0.053) |
| Being in a committed relationship(1) | -0.010 |
|  | (0.106) |
| Harshness * Family support | -0.086 |
|  | (0.061) |
| Unpredictability * Family support | -0.011 |
|  | (0.055) |
| Harshness * Poverty rate by municipality | -0.012 |
|  | (0.053) |
| Unpredictability * Poverty rate by municipality | -0.052 |
|  | (0.048) |
| Harshness * Being in a committed relationship(1) | 0.056 |
|  | (0.109) |
| Unpredictability * Being in a committed relationship(1) | -0.066 |
|  | (0.104) |
| Observations | 362 |
| R^2^ | 0.103 |
| Adjusted R^2^ | 0.067 |
| Residual Std. Error | 0.914 (df = 347) |
| *Note.* Similar to the previous table (Supplementary Table 4), the risk measure was obtained by standardizing the number of pumps across the 30 trials (in the case of Griskevicius et al., 2013, there were 10 trials), then, the number of explosions was standardized for each participant. Finally, both standardized measures were averaged. *p<0.05; **p<0.01; ***p<0.001. | |

**Robust Regression Models Using BART Total Explosions as Dependent Variable**

In our study, we also expanded our analytical scope by incorporating the total number of balloons exploded as a dependent variable. This additional measure augments the existing analytical framework by providing another dimension for examining risk behavior.

**Table S5.** BART Total Explosions Robust Regression Models for Hypothesis 1

|  | *Dependent variable* | | |
| --- | --- | --- | --- |
|  | Total Explosions Standardized | | |
|  | (1) | (2) | (3) |
| Constant | 0.29** | 0.28* | 0.10 |
|  | (0.11) | (0.11) | (0.13) |
| Sex(1) | -0.25* | -0.28* |  |
|  | (0.11) | (0.11) |  |
| Age | -0.08 | -0.13 | -0.21** |
|  | (0.06) | (0.07) | (0.08) |
| Context (1) | -0.36** | -0.31* | -0.48** |
|  | (0.13) | (0.13) | (0.17) |
| Harshness | 0.04 | 0.07 | 0.17* |
|  | (0.06) | (0.06) | (0.08) |
| Unpredictability | -0.04 | -0.02 | -0.04 |
|  | (0.05) | (0.06) | (0.07) |
| Age at first sexual relationship |  | 0.11* | 0.20** |
|  |  | (0.05) | (0.06) |
| Age at menarche |  |  | 0.11 |
|  |  |  | (0.09) |
| Harshness * Age at first sexual relationship |  | 0.03 | 0.09 |
|  |  | (0.06) | (0.06) |
| Unpredictability * Age at first sexual relationship |  | 0.02 | 0.08 |
|  |  | (0.06) | (0.05) |
| Harshness * Age at menarche |  |  | 0.02 |
|  |  |  | (0.08) |
| Unpredictability * Age at menarche |  |  | -0.11 |
|  |  |  | (0.07) |
| Observations | 368 | 330 | 173 |
| R^2^ | 0.07 | 0.08 | 0.20 |
| Adjusted R^2^ | 0.05 | 0.06 | 0.16 |
| Residual Std. Error | 0.98 (df = 362) | 0.96 (df = 321) | 0.90 (df = 162) |
| *Note.* The risk measure used was the total number of explosions. This variable was standardized to z-scores. *p<0.05; **p<0.01; ***p<0.001. | | | |

**Table S6.** BART Total Explosions Robust Regression Model for Hypothesis 2

|  | *Dependent variable* |
| --- | --- |
|  | Total Explosions Standardized |
| Constant | 0.29* |
|  | (0.12) |
| Sex(1) | -0.22* |
|  | (0.11) |
| Age | -0.07 |
|  | (0.06) |
| Context (1) | -0.32* |
|  | (0.13) |
| Harshness | 0.02 |
|  | (0.09) |
| Unpredictability | -0.02 |
|  | (0.09) |
| Family support | -0.05 |
|  | (0.06) |
| Poverty rate by municipality | -0.08 |
|  | (0.05) |
| Being in a committed relationship | -0.03 |
|  | (0.11) |
| Harshness * Family Support | -0.07 |
|  | (0.06) |
| Unpredictability * Family Support | 0.01 |
|  | (0.06) |
| Harshness * Poverty rate by municipality | -0.04 |
|  | (0.06) |
| Unpredictability * Poverty rate by municipality | -0.05 |
|  | (0.05) |
| Harshness * Being in a committed relationship | 0.08 |
|  | (0.11) |
| Unpredictability * Being in a committed relationship | -0.03 |
|  | (0.11) |
| Observations | 362 |
| R^2^ | 0.08 |
| Adjusted R^2^ | 0.05 |
| Residual Std. Error | 0.98 (df = 347) |
| *Note.* The risk measure used was the total number of explosions. This variable was standardized to z-scores. *p<0.05; **p<0.01; ***p<0.001. | |

**Section 3. Outliers**

The subsequent findings were conducted both as a diagnostic measure and to bolster the application of robust linear regression models. For this purpose, we utilized the variables incorporated within the linear regression model outlined in our second hypothesis, given that this model encompasses a broader range of parameters. Below (Figure S3), it’s possible to appreciate that there are some deviations from the straight line at both ends of the distribution. The lower left tail of the distribution deviates slightly from the line, and the upper right tail shows a more pronounced deviation, with several points turning off the expected line for a normal distribution. This could indicate that the residuals have heavier tails than expected under normality, suggesting the presence of possible outliers or a long-tailed distribution.


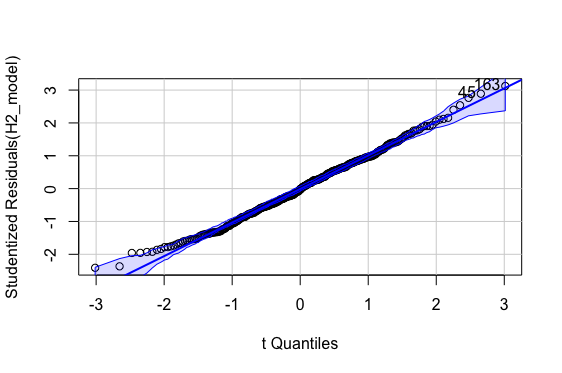


**Figure S3.** Hypothesis 2 QQ-Plot Regression Model

In Figure S4, it is possible to visualize the influence of individual data points on the regression model. This type of plot combines information on: (1) the leverage of each observation (as measured by hat-values), (2) the size of the residuals (standardized), and (3) the influence of observations (as measured by Cook's distance). The x-axis shows the hat-values, these values measure the leverage of each observation, which is an indication of how far an independent variable's value is from the mean of the independent variables. Observations with high leverage can have an outsized effect on the estimation of the regression coefficients. Furthermore, the dashed vertical lines typically represent potential thresholds for high leverage points. Observations to the right of these lines are considered to have high leverage. The horizontal dashed lines (at $\pm$ 2) are common cutoffs for identifying large residuals. Observations outside these lines are considered to have large residuals. The size of the circles is proportional to the Cook's distance of the observations. A larger circle indicates a greater influence on the regression model, meaning that omitting that data point would notably change the regression coefficients. Data points that are labeled with observation numbers are those that the plot function has automatically identified as being influential. They typically are points with high leverage, large residuals, or both. Furthermore, we test if those labeled data points have a real impact in the model. In this vein, we remove those rows points from our database and re-run our regression model. Overall, we found that there was an interaction between harshness and family support (β = -0.13, SE = 0.06, *p* = 0.04). Additionally, the adjusted R^2^ of the model increase from 0.058 to 0.068. Its important to mentioned that we only removed a few participants (five) in this analysis, in order to show the possible impacts of removing them in our model.


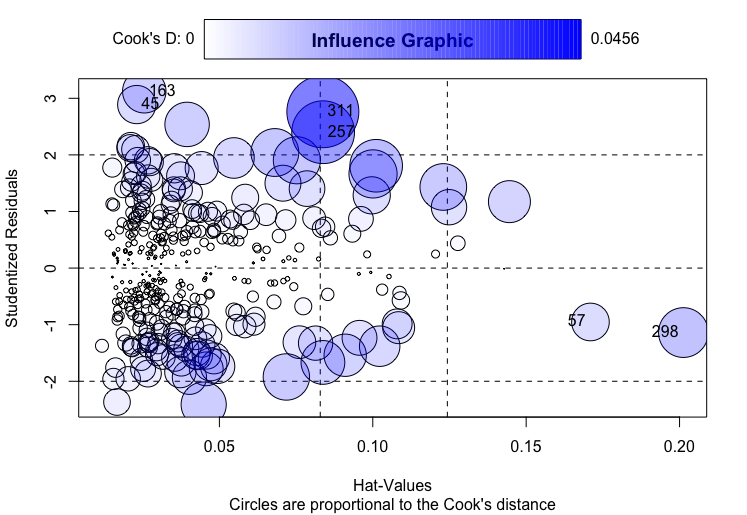


**Figure S4.** Hypothesis 2 Model Influence Graphic

Figure S5 shows the DFFITS values for each observation in the model. DFFITS shows the influence of each observation on the fitted values. Specifically, it measures the change in the predicted value for an observation when that observation is left out of the model. The threshold lines (which were calculated by $\pm2 \sqrt{p/n}$ (where p is the number of parameters in the model, including the intercept, and n is the number of observations), are used to identify observations that have a large influence on the model. Observations with DFFITS values outside of these lines are considered to be influential. In the figure there are a few observations with DFFITS values that exceed the threshold, which are potential points of concern.


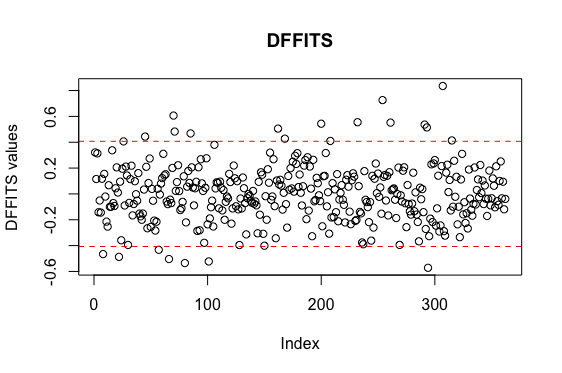


**Figure S5**

*Hypothesis 2 Model DFFITS*

Table S7 shows the frequency of DFBETAS outside $\pm$ 2 (a common threshold of DFBETAS that have small to moderate influence on the coefficient estimate) for each predictor (including the intercept) of hypothesis 2 regression model. Specifically, DFBETAS is a measure of the influence of an individual observation on the parameter estimate (the estimated coefficient) of each predictor in the regression model.

**Table S7**

Frequency of DFBETAS Outside $\pm$ 2 Threshold

| Predictors of hypothesis 2 | DFBETAS (outside threshold) frequency |
| --- | --- |
| (Intercept) | 3 |
| Harshness | 4 |
| Family support | 4 |
| Unpredictability | 4 |
| Poverty rate by municipality | 3 |
| Being in a committed relationship | 2 |
| Sex | 0 |
| Age | 3 |
| Context | 1 |
| Harshness * Family support | 8 |
| Unpredictability * Family support | 4 |
| Harshness * Poverty rate by municipality | 6 |
| Unpredictability * Poverty rate by municipality | 5 |
| Harshness * Being in a committed relationship | 3 |
| Unpredictability * Being in a committed relationship | 5 |

^Note.^

Supplementary Figure 4 shows the COVRATIO values in the regression model. They are an indicator of the influence of an observation on the precision of the coefficient estimates. More specifically, COVRATIO measures the impact of deleting an observation on the covariance matrix of the coefficient estimates. A COVRATIO value close to 1 (red line) indicates that the observation has a typical influence on the covariance matrix. Values significantly greater than 1 indicate that the observation increases the precision of the coefficient estimates, while values significantly less than 1 indicate that the observation decreases the precision. In the figure, a few observations have COVRATIO values that deviate from 1, indicating they might be influential.


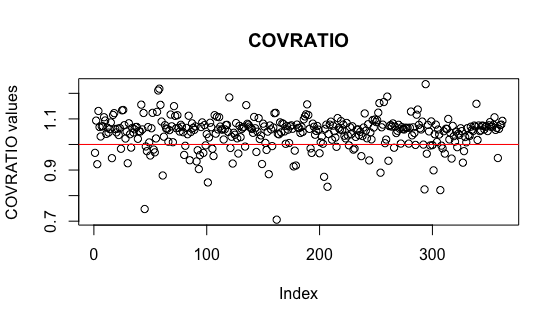


**Figure S6**

*Hypothesis 2 Model COVRATIOS*
